# African leadership is critical in responding to public health threats

**DOI:** 10.1038/s41467-024-45220-3

**Published:** 2024-01-29

**Authors:** Nicaise Ndembi, Aggrey Aluso, Mahlet K. Habtemariam, Landry Tsague, Gwen Mwaba, Abdu Muktar, Nafisa Jiwani, Krishna Udayakumar, Trevor A. Crowell, Alain Ngashi Ngongo, Jean Kaseya

**Affiliations:** 1 Africa Centres for Disease Control and Prevention (Africa CDC), Addis Ababa, Ethiopia; 2 Institute of Human Virology, University of Maryland School of Medicine, Baltimore, Maryland USA; 3 Pandemic Action Network, Resilience Action Network Africa, Nairobi, Kenya; 4 African Export-Import (Afrexim) Bank, Cairo, Egypt; 5 African Development Bank (AfDB), Abidjan, Côte d’Ivoire; 6 U.S. International Development Finance Corporation (DFC), Washington, District of Columbia USA; 7 Duke Global Health Innovation Center, Durham, NC USA; 8 Henry M. Jackson Foundation for the Advancement of Military Medicine, Bethesda, Maryland USA

**Keywords:** Government, Diseases, Public health, Developing world

## Abstract

The African continent demonstrated decisive leadership throughout its response to the COVID-19 pandemic, leveraging lessons learned from previous outbreaks and acting quickly to limit the impact of the SARS-CoV-2 virus. We propose a framework to build on these successes that calls for greater collaboration between African leaders, and greater inclusion of African voices in the global health ecosystem.

Inequities—both social and economic—drive pandemics, and lead to unnecessary loss of life, devastating economic consequences, and declining trust in science and public health policies^1–3^. Like much of the world, Africa is recovering from the COVID-19 pandemic during which it experienced delayed access to life-saving vaccinations and therapeutics, which were a direct result of global inequities in resource sharing. Despite these challenges, Africa did successfully structure and implement its own procurement mechanism for 400 million doses of J&J COVID-19 vaccines in 2021, which was implemented through the African Vaccine Acquisition Trust (AVAT), Africa Medical Supply Platform (AMSP) in collaboration with Afreximbank and the Africa Centres for Disease Control and Prevention (Africa CDC). This was made possible by collaboration among African institutions under the leadership of African Union Heads of State and Government^3^. This collaboration resulted in the creation a single Africa-wide AVAT No Fault Compensation Scheme for African Union Participating Member States, which provided eligible vaccine recipients with access to prompt, fair and transparent compensation for serious adverse events associated with COVID-19 vaccinations.

The success of the African Union vaccine procurement scheme emphasizes the importance of collaboration among African institutions. Inspired by this and by Africa CDC’s New Public Health Order of the African Union, we propose building an African coalition of support within the Intergovernmental Negotiating Body of the World Health Organization for a Pandemic Accord process. To achieve this, we propose a framework of three principles and call on all African leaders to unite behind them.

First, we need a new framework that puts human rights and shared public interest at the heart of global health security decisions. Pandemics pose existential threats to economies, the achievement of the Sustainable Development Goals (SDGs), and to humanity’s collective security and survival. Without putting the protection of people, families, and societies at the heart of our global health security decisions, we will simply entrench a status quo that is defined by inequity. Recently, Member States of the World Health Organization (WHO) agreed to a global and collaborative process to draft an international instrument to strengthen pandemic prevention, preparedness and response^4–6^. These Pandemic Accord negotiations offer an opportunity to put the human right to survival at the centre of a new global compact—we owe it to those that lost their lives, their livelihoods, their mental health, and to those that could not access tests, treatments, or vaccines to make an explicit framework that reasserts the right for lives to be treated equally, wherever someone is born.

Second, African leaders must participate in global health security governance processes as equal stakeholders. In May 2023, Africa CDC and WHO announced the launch of the Joint Emergency Preparedness and Response Action Plan (JEAP). The JEAP underscores the shared vision to strengthen the continent’s emergency preparedness and response and health systems. JEAP is a pioneering five-year strategic collaboration to boost emergency preparedness and response efforts throughout the continent to ensure that disease outbreaks are managed efficiently. The JEAP strengthens emergency preparedness and response across five priority collaboration areas: (i) Country assessments in the preparedness context; (ii) Workforce development; (iii) Surveillance, including diagnostics and genome sequencing; (iv) Logistics, supply chain and stockpiling; and (v) Response readiness and coordination. The plan builds upon existing frameworks and initiatives while capitalizing on the Africa CDC and WHO’s unique capabilities and resources.

Third, the Access to COVID-19 Tools Accelerator (ACT-A) exposed significant gaps in the current global health financing and governance systems; in Africa, it highlighted the need to move beyond charity-driven donation models for life-saving therapies, and instead move to sustainable and equitable finance models for local vaccine, diagnostics and therapeutics manufacturing. Africa CDC has implemented a Partnerships for African Vaccine Manufacturing (PAVM), which aims to leverage pan-African and global partnerships to scale-up vaccine manufacturing on the continent to meet the needs of 60% of African Union countries^7^. Currently vaccine manufacturing in Africa can only meet 1% of demand on the continent^7^. Africa’s vaccine need represents 25% of the global volumes with an estimate of 1.5 billion doses annually and expectations to surpass 2.7 billion doses by 2040^8,9^.

Africa’s investment in its own health security extends further as the African Developmental Bank has also supported continental efforts to build institutional capacity at regional and country levels, which has improved pharmaceutical harmonization. Furthermore, the Bank is working closely with African Union/New Partnership for Africa’s Development (NEPAD) in developing a full support package for the establishment and early operations of the African Medicines Agency^10^, and it has established the African Pharmaceutical Technology Foundation in Kigali, to foster technology transfer, skills development, and Research and Development capabilities. AMA will be established as a Specialized Agency of the African Union (AU) dedicated to improving access to quality, safe and efficacious medical products in Africa. Finally, the Bank is part of a group of lenders financing four pioneering vaccine development projects for routine immunization in Senegal, Egypt, Ghana and South Africa, which will begin in 2024.

All these projects collectively demonstrate the importance of African-funded projects to meet the continent’s health demands^11,12^. The projects discussed here all aim to shift the conversation about the donation to Africa of spare life-saving therapies and diagnostic tests, which are produced in high-income countries, to how best to create resilient health systems in Africa. The focus will, therefore, shift to identifying facilities that require investment and financing, technology transfer, human capital development, regulatory support, and models for financial sustainability in the event of health security threats.

Globally, Africa’s lessons, expertise and leadership can and must be utilized to address broader health challenges, which is most recently demonstrated by the success of Egypt, a member of the African Union, in achieving elimination of viral hepatitis C from 10% in 2008 to 0.38 % in 2022. In October 2023, Egypt achieved the Gold Tier on the path to eliminating hepatitis C, making it the first country to achieve this worldwide recognition. As global leaders come together to improve pandemic preparedness, prevention and response, the urgency needed to deliver optimized health for all people, and the planet, must be addressed. Strengthening voices from the African Union on the world stage is critical for ensuring the inclusion of **African** citizens as equal members of the global community.
